# When Did Coloring Books Become Mindful? Exploring the Effectiveness of a Novel Method of Mindfulness-Guided Instructions for Coloring Books to Increase Mindfulness and Decrease Anxiety

**DOI:** 10.3389/fpsyg.2018.00056

**Published:** 2018-01-30

**Authors:** Michail Mantzios, Kyriaki Giannou

**Affiliations:** ^1^Department of Psychology, Birmingham City University, Birmingham, United Kingdom; ^2^Division of Neuroscience and Experimental Psychology, School of Biological Sciences, University of Manchester, Manchester, United Kingdom

**Keywords:** mindfulness, coloring books, anxiety, mandala, Mindfully-Guided Coloring Books (MGCB)

## Abstract

Mindfulness has been associated with the use of coloring books for adults; however, the question of whether they do increase mindfulness has not been addressed. In two studies, we attempted to identify whether mindfulness is increased, and whether there is a need for ongoing guidance while coloring, similar to mindfulness meditation. In the first randomized controlled experiment, university students (*n* = 88) were assigned to an unguided mandala coloring group (i.e., described in mainstream literature as a mindfulness practice) or to a free-drawing group. Measurements of state mindfulness and state anxiety were taken pre- and post- experiment. Results indicated no change in mindfulness or anxiety. In the second randomized controlled experiment, university students (*n* = 72) were assigned to an unguided mandala coloring group (i.e., same as Experiment 1), or, to a mindfulness-guided coloring group (i.e., same as the unguided coloring group with a mindfulness practitioner guiding participants as in mindfulness breathing meditation, with instructions modified and applied to coloring). Results indicated that the mindfulness-guided mandala coloring group performed better in decreasing anxiety, but no change was observed in mindfulness. Exit interviews revealed that some participants did not like the voice guiding them while coloring, which suggested further differing and significant findings. While mindfulness-guided coloring appears promising, guidance or instructions on how to color mindfully may require further development and adjustment to enhance health and wellbeing.

## Introduction

Mindfulness is a new element incorporated in coloring books, with their popularity among book publishers and consumers rapidly increasing (Halzack, 2016). Kabat-Zinn (1990) described mindfulness as an awareness that emerges through purposefully paying attention in the present moment, non-judgmentally. Similarly, Bishop et al. (2004) described mindfulness as the self-regulation of attention in an effort to achieve a non-elaborative awareness of the current experience. Both definitions are more aligned to mindfulness practices, which are used to cultivate the ability to be attentive and aware of the present moment in a non-evaluative way. Mindfulness practices, such as breathing meditation, entail concentrating fully on one aspect of the present moment; in this case, the breath. Observations of attention shifting away from the breath into thoughts, feelings and emotions are acknowledged and accepted (e.g., by labeling a thought such as “I think I am a failure” as a “thought”), and re-focusing on the breath is something that is happening repeatedly, which briefly entails a cycle of practice. Understanding that the mind does this from time to time, and enabling simple observation and non-reaction to such experiences, while simply turning the attention to the breath enables the self-regulation of attention. Practices include meditative and non-meditative practices (e.g., Mantzios and Wilson, 2014; Hanley et al., 2015), all of which are accompanied by general instructions and ongoing guidance prior and/or during practices. In fact, mindfulness cannot be practiced without general instructions and ongoing guidance when people do not have any prior experience, while coloring books appear to have minimal to no instructions and/or guidance. Many coloring books are proclaimed as “mindful” without the usual guidance that is observed in mindfulness practices, and contemporary peer-reviewed research appears to do the same in declaring something that has not been scientifically proven (e.g., Carsley et al., 2015).

Mindfulness has enjoyed a remarkable increase in research and popularity in the past decade (Shapiro and Carlson, 2009), and this may be the reason for trying to increase sales of coloring books. Without wanting to take away the Sanskrit origin and Tibetan thought and practice surrounding the use of coloring mandalas, the very basic elements of mindfulness appear to be absent from contemporary coloring books and research practices.

As previously mentioned, Kabat-Zinn (1990) described mindfulness as an awareness that emerges through purposefully paying attention in the present moment, non-judgmentally. People may benefit from practicing mindfulness in reducing levels of anxiety (Anderson et al., 2007), depression, fatigue, health-related quality of life (Grossman et al., 2010), rumination (Jain et al., 2007), and stress (Shapiro et al., 2005). In order to achieve the benefits of mindfulness training, one needs to wilfully keep awareness focused on whatever is present, without fixating on any particular part of that experience, or engaging in any secondary processing (Goleman, 1988). This description of mindfulness shows that there are ways of transforming any experience into a mindful one (e.g., Hanley et al., 2015), but at the same time, without accurate instructions and guidance, an experience can become mindless, fixated, and avoidant. Mindfulness helps people face, endure and accept the present moment (and not avoid it), which may not be the case with coloring books.

There is great variation in coloring books that are suggested to increase mindfulness. Some of those mindfulness coloring books have rectangular shapes to color in, while other books include circular shapes. We also noted that a minority of coloring books come with relaxation sounds (e.g., nature sounds), while other books are not accompanied by complementary audio files. Furthermore, some books come with generic instructions of what the book does and not how to color (e.g., this mindfulness coloring book will help you relax), which may well introduce the possibility of priming or of a placebo effect, while other books are not accompanied by any instructions. Considering the popularity of coloring books, there are clear implications for people who are willing to incorporate mindfulness into their lives with the alternative mean of coloring books (instead of meditation), and demands research of directing public health and well-being. The initial question that is addressed in the current research is whether the basic unguided mandala coloring books (as they exist and are used by consumers) increase mindfulness and decrease anxiety.

Coloring books and the use of mandalas (i.e., circular art shapes) have been held as a method of alleviating stress and anxiety, improving mood and physiological changes in past and contemporary art therapy literature. For example, DeLue (1999) investigated the effect of coloring mandalas on relaxation by recording physiological changes, such as heart rate. The study found a decrease in heart rate, and provided some preliminary results on the effect of coloring mandalas and physiological health.

The majority of the literature, however, has been enthusiastic in investigating psychological health. Curry and Kasser (2005) explored the effect of coloring mandalas on anxiety in adults. They assigned students to a mandala, plaid (i.e., a checkered design) or free-drawing group. Results indicated a significant decrease in anxiety levels for both the mandala and plaid groups, while the free-drawing group showed no change between pre- and post- measurements. Curry and Kasser (2005) were the first authors to suggest an association between mindfulness and coloring books, but this association was only mentioned in the introduction of their manuscript and was not the topic of the research itself. In other words, their research did instigate, but not investigate mindfulness.

van der Vennet and Serice (2012) replicated Curry and Kasser’s (2005) study, and found that the mandala group reduced anxiety levels significantly more than the plaid and free-drawing groups. Interestingly, contrary to Curry and Kasser’s findings, the free-drawing group, although not statistically significant, outperformed the plaid group in reducing anxiety. Despite these mixed findings, recently published coloring books of a plaid design are described as mindful coloring books (e.g., books published by Harper Collins Publishers), which is inconsistent with previous findings.

On the other hand, there is a Jungian movement, where methodology and theory around mandalas is about drawing in a circle, rather than to coloring in an abstract design. These mandalas entail mechanisms such as having an active imagination, self-reflection, an integration between conscious and unconscious (see Potash et al., 2015 for a brief review), which are far from the underlying mechanisms of mindfulness practices. Henderson et al. (2007) worked with Jungian mandalas to explore the longitudinally effect of coloring mandalas (vs. a written disclosure paradigm) for people suffering from Post-Traumatic Stress Disorder (PTSD). Among the outcome measures were stress, anxiety, and PTSD symptoms. Results revealed that only the PTSD symptoms significantly decreased after a 1-month follow-up. The mandala used in this study, however, was an unstructured mandala, which consisted of a circle to draw in (instead of a design to color in), and cannot be directly compared to other findings reported in this review of coloring book literature and scientific research. Babouchkina and Robbins (2015) used a similar rationale, employing a square or circle design, with (and without) instructions of expressing one’s feelings while drawing, creating overall four separate conditions in their experiment. Mood enhancement was observed only in circular conditions, and supported the Jungian rationale on the effectiveness of circular coloring and drawing. Overall, Curry and Kasser (2005) and Carsley et al. (2015) speculated a mindfulness rationale and theory, but both did not attempt to investigate the relationship between mandala coloring books and mindfulness. Overall, any claims around mindfulness coloring books have no scientific bearing, considering that no research has been conducted to investigate whether any type of coloring increases mindfulness.

In summary, findings indicate that the use of coloring books assists in reducing anxiety and in improving mood (Curry and Kasser, 2005; Babouchkina and Robbins, 2015), but other results suggest such findings to be unclear (van der Vennet and Serice, 2012; Carsley et al., 2015), while some coloring books take a Jungian approach (Potash et al., 2015) that is quite diverse to mindfulness theory and practice; all of which create a need for further exploration. Therefore, this research first set forth an exploration whether mandala coloring (vs. free-drawing) significantly increased state mindfulness and decreased state anxiety.

## Experiment 1

As a proof-of-concept to the critical and contradictory literature surrounding coloring books, the initial exploration assessed whether mandala coloring, which is suggested to be a mindfulness practice, increased mindfulness and decreased anxiety above a random art activity that related to freely drawing on a piece of paper.

### Method

#### Participants

Eighty-eight female (*n* = 44) and male (*n* = 40) undergraduate students were recruited from a University located in the West-Midlands of the United Kingdom. The age of participants ranged from 18 to 26 years (*M* = 21.32, *SD* = 1.54). Four participants were excluded from the final analyses (further details can be found in the procedure section below). The ethnicity distribution was as follows: 71.4% (*n* = 60) of participants stated Caucasian, 14.3% (*n* = 12) as South East Asian, 1.2% (*n* = 1) as Black, 8.3% as Indian (*n* = 7), and 4.8% as Mixed (*n* = 4). Three participants reported that they were currently using coloring books, and two described infrequent use (i.e., few times throughout the week), while one reported daily use. Participants were recruited on a voluntary basis and did not participate for any course credits or financial reward. A funneled debriefing procedure led to the exclusion of four participants from the final analysis, which is described in more detail within the procedure section.

#### Materials

##### Participant information form

This form asked for the participants’ age, gender, ethnicity, past and current usage of coloring books, and the frequency of usage. Please note that the age [*t*(82) = 0.97, *p* = 0.33] and frequency of use of coloring books [*t*(17) = 0.45, *p* = 0.66] did not significantly differ between the experimental and control groups.

##### State Anxiety Inventory, Short-Form (SAI; Marteau and Bekker, 1992)

State anxiety was assessed with the six item short-form SAI scale. Responses range from 1 (*not at all*) to 5 (*very much*) and are summed up to calculate a total score. Higher scores reflect higher levels of anxiety. Sample items are “I am tense” and “I feel calm.” The use of the short-form related to enhancing engagement and avoiding boredom, which may be cases for further investigation when investigating samples that are naïve to the experimental condition. The present study produced an alpha of 0.78/0.79 for pre- and post- measurements.

##### State Mindfulness Scale (SMS; Tanay and Bernstein, 2013)

The State Mindfulness Scale is a state-like measurement tool that includes 21 items, with responses ranging from 1 (*not at all*) to 5 (*very well*). The SMS measure consists of two sub-scales that relate to bodily sensations or mental events. Sample items are “I noticed some pleasant and unpleasant physical sensations” and “I noticed emotions come and go.” Higher scores reflect greater levels of state-mindfulness. This scale was judged to be more inclusive of present moment awareness and a non-judgmental attitude, compared to the alternative scale that is evaluating mindful attention and awareness of the present moment (see Brown and Ryan, 2003 for alternative scale). The present study produced an alpha of 0.95/0.94 for pre- and post- measurements of the overall score; and 0.82/0.85 for the bodily sensations and 0.94/0.93 for the mental events subscales.

##### Coloring material

Both groups were exposed to an A4 size page, and the page was either blank (for the free-drawing group) or with a mandala design (see Freeman, 2016). All participants were exposed to 10 colored pencils, in a transparent cup.

#### Procedure and Design

Potential participants responded to an advertisement at a University in the West Midlands of the United Kingdom. Participants were kept blind to the study, and were informed that they signed-up to volunteer at a study that was investigating “Personality and Art.” Pre-screening questions to allow participation evaluated color-blindness, medication use, and former and current diagnoses of affective disorders. Participants who fulfilled the criteria were randomly assigned either to the ‘mandala’ or ‘free-drawing’ condition. They received simultaneously a participant information form, a consent form, and the demographics page with the questionnaire (i.e., the two scales) for the pre-assessment. Next, participants commenced a 10-min coloring or freely drawing depending on the condition they were assigned to. The authors note that prior pilot work by the authors of this manuscript comparing 10- vs. 20-min coloring showed no significant differences in both anxiety reduction and increase in mindfulness. Their aim was to develop interventions that are shorter, easily implemented and adhered to by clinical and non-clinical populations in the future. Also, other research comparing 10- vs. 20-min mindfulness practices showed no significant differences in both stress reduction and increase in mindfulness (see Berghoff et al., 2017). After the 10 min, participants were given the two questionnaires again for the post-assessment, and were debriefed and thanked for their participation. A funneled debriefing procedure was followed, where participants were asked whether they were aware of the aim of the study, what outcome was investigated, and if they realized that the questions were the same between pre- and post- measurements. The funneled debriefing procedure led to three exclusions, where participants expressed a full awareness of experimental procedures and identified the aim, scales, and outcome measures of the experiment. Other information was obtained as well through the funneled debriefing, such as whether the participants were meditating or had any knowledge around mindfulness, or any other involvement in stress or anxiety reduction programs. Those three participants also claimed knowledge around mindfulness meditation and experience in practicing, knowing that the lead researcher is a mindfulness expert, and the experiment involving mindfulness coloring books. Aiming to recruit participants blind to the experimental condition and naïve to mindfulness exercises, the funneled debriefing assisted in having a homogeneous sample across the two groups. An additional participant was excluded on grounds of speed (i.e., filling in 31 items in less than 1 min and later analyses revealing that reverse scored items were given similar values to positively scored items of the same scale). Participants had the opportunity to record an arbitrary number assigned to their questionnaires and drawing, to allow them to withdraw at a later stage and retain the anonymity of participation. Ethical approval was granted by the Ethical Committee based within the University and was scrutinized to strictly adhere to ethical guidelines set by the British Psychological Society.

Data were analyzed by utilizing two 2 × 2 mixed ANOVAs, and the sample size recruited matched or exceeded previous studies. Analyses were conducted by utilizing SPSS version 22 (IBM, 2013) and a significance threshold was set at *p* < 0.05.

### Results

Two 2(Group Type: Mandala, Free-drawing) × 2(Time: Pre, Post) ANOVAs with repeated measures on the Time was conducted on the Anxiety and Mindfulness scales.

For Mindfulness, there was no significant main effect of Time: *F*(1,82) = 2.75, *p* = 0.10, , and there was no significant main effect of Group Type: *F*(1,82) = 0.40, *p* = 0.85 (see **Table 1**). There was also no significant interaction between Time and Group Type, *F*(1,82) = 2.34, *p* = 0.13. Please note that the same design was used to explore the mind and body subscales, but the results were equally non-significant.

**Table 1:** Means and standard deviations for the Mandala group (*n* = 40) and free-drawing group (*n* = 44), pre- and post-intervention.

		Mean (*SD*)		
	Measures	Pre	Post	Post–Pre
Mandala	SAI	12.30 (4.51)	11.42 (4.36)	-0.88 (3.17)
	SMS	71.42 (18.67)	71.70 (18.67)	0.28 (20.26)
Free-drawing	SAI	12.70 (3.96)	11.20 (4.18)	-1.50 (3.23)
	SMS	67.54 (17.21)	74.34 (16.36)	6.80 (18.79)

*SMS, State Mindfulness Scale; SAI, State Anxiety Scale*.

For Anxiety, there was a significant main effect of Time: *F*(1,82) = 20.67, *p* < 0.001, $\eta_{p}^{2}$ = 0.20, with both groups decreasing over time in their Anxiety scores (see **Table 1**). There was no significant main effect of Group Type, *F*(1,82) = 0.002, *p* = 0.98. Also, no significant interaction was found between Time and Group Type, *F*(1,82) = 0.96, *p* = 0.33. Results indicated that coloring produced a decrease in anxiety levels, but both groups produced similar outcomes.

### Discussion

The first experiment suggests that there are no significant differences between mandala coloring and free-drawing in reducing anxiety. Furthermore, no significant differences between mandala coloring and free-drawing were found in state mindfulness levels. Coloring books could be a useful mindful tool, especially when considering their popularity, but only with the right guidance and intentions. The intention to be attentive and aware of the present moment can be achieved, but whether the process will involve acceptance and non-judgment, or, avoidance and rumination, is questionable. Instructions on how to color mindfully, and ongoing guidance during coloring that assimilates the instructions that are usually given during mindfulness meditation may well be a method of increasing mindfulness using coloring books.

## Experiment 2

### Introduction

Experiment one suggests the integration of mindfulness guidance with coloring, to attempt to develop a mindfulness coloring experience that is more consistent with meditative practices. The present research compared unguided and mindfulness-guided coloring against changes in state mindfulness and anxiety. Mindfulness meditation entails guidance, and is one of the vital elements that are enabling novice meditators to enhance their practice (Kabat-Zinn, 1990).

The practice is understood more simply as attentional training, a process of consciously keeping one’s awareness focused on whatever is present, without fixating on any particular part of that experience (Goleman, 1988). *Ânâpânasati* (i.e., the development of mindfulness of breathing), is a core contemplative practice in Buddhism, and the foundation of secular practices in the Western world. Breathing becomes the primary focus to keep attention to the present moment, and allows for a natural observation of being. During observations of being in the moment, shifts of attention away from the breath and into thoughts, feelings and/or emotions allows the meditator to non-reactively and non-judgmentally return to the present moment experience by accepting or acknowledging that the mind does this from time to time. This nature of meditative practice may be expressed in a many ways, from mindfulness breathing meditation (Kabat-Zinn, 1990) to informal sessions of walking mindfully (e.g., Kabat-Zinn, 1990; Levine, 2007).

But informal practices can vary, and depend on the present occurrences and behavioral enactments such as walking into work would be best practiced through mindful walking. Mindfulness teachers emphasize the importance of everyday mindfulness, and suggest any chance to practice mindfulness should be taken, as each present moment represents a unique moment in time to step outside the ‘doing’ and into the ‘being’ mode. Whilst this has not been the case with coloring books, aligning mindfulness meditative instructions to the practice of coloring as attempted in this experiment may well be a novel manifestation of genuine “Mindfulness Coloring Books.” We are expecting that mindfully guided coloring would significantly outperform the unguided coloring in increasing mindfulness and decreasing anxiety.

### Method

#### Participants

Seventy two (female, *n* = 68) undergraduate students were recruited from a University located in the West-Midlands of the United Kingdom. The age of participants ranged from 18 to 51 years (*M* = 22.52, *SD* = 6.29). The ethnicity distribution was as follows: 55.6% (*n* = 40) of participants stated Caucasian, 16.7% (*n* = 12) as South East Asian, 9.7% (*n* = 7) as Black, 5.6% as Indian (*n* = 4), 11.1% as Mixed (*n* = 8), and 1.4% as Chinese (*n* = 1). Participants were recruited on a voluntary credit reward system within the School of Sciences.

#### Materials

##### Participant information form

This form was the same as in Experiment 1. Please refer to previous description. Please note that the age [*t*(69) = -0.29, *p* = 0.77] and frequency of use of coloring books [*t*(30) = -0.62, *p* = 0.53] did not significantly differ between the experimental and control groups.

##### State Anxiety Inventory, Short-Form (SAI; Marteau and Bekker, 1992)

Please refer to Experiment 1 for full description of the scale. The present study produced an alpha of 0.88/0.91 for pre- and post- measurements.

##### State Mindfulness Scale (SMS; Tanay and Bernstein, 2013)

Please refer to Experiment 1 for full description of the scale. The present study produced an alpha of 0.92/0.94 for pre- and post- measurements of the overall score.

##### Coloring material

Both groups were exposed to an A4 size page with a mandala design (see Freeman, 2016). All participants were exposed to 10 colored pencils placed in a transparent cup.

##### Mindfulness coloring instructions

Guidance used in a typical mindfulness breathing meditation was adjusted to fit into the coloring activity. The same meditation teacher was present at each experimental session and used a specific protocol and times of silence for each session within the 10-min coloring exercise to keep the practice consistent between practices. **Table 2** has exemplars from the coloring protocol used, and the full transcript is obtainable by contacting the first author.

**Table 2:** Sample sentences from the protocol that was used for the mindfulness-guided coloring by the meditation teacher.

Beginning	“Let your entire body relax and lay your hands down on the table and direct your attention on the drawing and the colors.”	Initial concentration on the coloring page.
Middle	“Try to consciously observe the color transferring on the paper, the sensation of the pencil in your hand, your body sitting in the chair. Be aware of what happens moment to moment.”	Concentration on the coloring
Middle	“From time to time, your mind will wander onto the past or the future. If thoughts come up simply observe and describe them as ‘thoughts,’ and emotions as ‘emotions,’ without judging or evaluating whether they are ‘good’ or ‘bad.’ If this happens, remember to gently return to the present sensation of being aware of your coloring.”	Non-judgment/acceptance
End	“When you feel ready, slightly and gently refocus your eyes and allow yourself to slowly come back to the environment around you. Allow yourself some time and maintain in your sitting position for a few more seconds.”	Gentle and calm closing of the coloring session

#### Procedure and Design

Please refer to Experiment 1 for full description of procedure, which was followed for this experiment as well. Differences from Experiment 1 related to the comparison between Guided and Unguided mandala coloring conditions (see section “Materials”). The funneled debriefing was the same, but included one additional open ended question: (1) Did you like the guidance you received during the coloring activity? (used in the guided-coloring condition only). The funneled debriefing did not lead to any exclusions.

Data were analyzed by utilizing two 2 × 2 mixed ANOVAs, and the sample size recruited matched or exceeded previous studies.

### Results

Two 2(Mandala: Guided, Unguided) × 2(Time: Pre, Post) ANOVAs with repeated measures on the time was conducted on the Anxiety and Mindfulness scales.

For Mindfulness, there was no significant main effect of Time: *F*(1,70) = 0.21, *p* = 0.65, and there was no significant main effect of Group Type: *F*(1,70) = 0.49, *p* = 0.49. There was also no significant interaction between Time and Group Type, *F*(1,70) = 0.91, *p* = 0.34. Please note that the same analyses were used to explore the mind and body subscales, but the results were similarly non-significant. Despite the non-significant differences, the directionality of change was different, with unguided coloring decreasing mindfulness, while mindfulness-guided coloring increasing mindfulness (see **Table 2**).

For Anxiety, there was no significant main effect of Time: *F*(1,70) = 2.57, *p* = 0.11. There was also no significant main effect of Group Type, *F*(1,70) = 0.51, *p* = 0.48. However, a significant interaction was found between Time and Group Type, *F*(1,70) = 9.82, *p* = 0.003, partial $\eta_{p}^{2}$ = 0.13. *Post hoc* paired sample *t*-tests revealed that anxiety significantly decreased in the guided-coloring condition, *t*(35) = 3.06, *p* = 0.004, *d* = 0.20, which was not observed in the unguided coloring condition, *t*(35) = 1.22, *p* = 0.23. Results indicated that guided coloring produced a decrease in anxiety levels, while for the unguided group anxiety levels increased (see **Table 3**).

**Table 3:** Means and standard deviations for the mindfully guided (*n* = 36) and unguided (*n* = 36) groups, pre- and post-intervention.

		Mean (*SD*)		
	Measures	Pre	Post	Post–Pre
Guided	SAI	11.94 (4.27)	9.39 (3.51)	-2.56 (5.00)
	SMS	73.31 (12.35)	82.75 (15.83)	3.44 (18.40)
Unguided	SAI	10.75 (4.34)	11.69 (4.73)	0.83 (4.03)
	SMS	79.53 (17.99)	78.34 (18.50)	-1.19 (22.64)

*SMS, State Mindfulness Scale; SAI, State Anxiety Scale*.

Further explorations revealed that both interventions were not uniformly assisting participants in increasing mindfulness, χχ^2^(1, *n* = 72) = 0.22, *p* = 0.64, and decreasing anxiety, χ^2^(1, *n* = 72) = 3.79, *p* = 0.05. Using the values obtained from the differences between pre- and post- measurements, the data suggest that increasing levels of mindfulness is similar amongst the two interventions, while with state anxiety, approximately 20% more participants benefited from becoming less anxious in the mindfully guided group (see **Table 4**).

**Table 4:** Frequencies and percentages of increase and decrease in anxiety and mindfulness, for the guided group (*n* = 36), and the unguided group (*n* = 36).

	State Anxiety				State Mindfulness			
	Guided		Unguided		Guided		Unguided	
	*n*	Percent	*n*	Percent	*n*	Percent	*n*	Percent
Decrease	30	83,3	23	63,9	20	55,6	18	50,0
Increase	6	16,7	13	37,1	16	44,4	18	50,0

After the main analyses, other data collected during the demographic stage were tested as covariates, or, for the possibility of individual differences effecting the outcomes of the study. None of these variables significantly mediated the results, or proposed a significant difference between groups. For example, age was tested as a covariate and ethnic background was explored. Only one element proposed further insight into the intervention developed. During the debriefing, participants were asked whether they liked or disliked the researcher sitting in the background and narrate or instruct them how to color. Seventeen participants responded that they liked the idea of being reminded how to color, while nineteen disliked it and thought that it was ‘taking them out of the zone,’ found it distracting, or thought it was awkward. To explore whether having a preference or not of the mindfulness instructions over coloring dictated further analyses on both state mindfulness and anxiety.

An additional two 2(Guided Mandala: Liked, Disliked) × 2(Time: Pre, Post) ANOVAs with repeated measures on the time was conducted on the Anxiety and Mindfulness scales.

For Mindfulness, there was no significant main effect of Time: *F*(1,34) = 3.83, *p* = 0.059, and there was a significant main effect of Group Type: *F*(1,34) = 4.82, *p* = 0.035, $\eta_{p}^{2}$ = 0.124. There was also a significant interaction between Time and Group Type, *F*(1,34) = 38.35, *p* < 0.001, $\eta_{p}^{2}$ = 0.53.

*Post hoc* paired sample *t*-tests revealed that mindfulness significantly increased in the group who liked the guided-coloring, *t*(16) = 6.32, *p* < 0.001, *d* = 1.53, which was observed in the group which did not like the coloring guidance, *t*(18) = -2.87, *p* = 0.01, *d* = 0.64. Results indicate that liking mindfulness-guided coloring significantly increased mindfulness over disliking mindfulness instructions.

For Anxiety, there was a significant main effect of Time: *F*(1,34) = 9.27, *p* = 0.004, $\eta_{p}^{2}$ = 0.214. There was also no significant main effect of Group Type, *F*(1,34) = 3.29, *p* = 0.078. Also, no significant interaction was found between Time and Group Type, *F*(1,34) = 0.134, *p* = 0.71. Results indicated that guided coloring produced a significant decrease in anxiety levels for both conditions, while liking the mindfulness guidance throughout coloring enabled more anxiety reduction (see **Table 5**).

**Table 5:** Means and standard deviations for liking (*n* = 17) and disliking (*n* = 19) the mindfully guided practice, pre- and post-intervention.

		Mean (*SD*)		
	Measures	Pre	Post	Post–Pre
Liked Mindfulness	SAI	11.18 (4.38)	8.29 (3.47)	-2.88 (5.21)
Guidance	SMS	76.29 (11.96)	93.71 (11.60)	17.41 (11.36)
Disliked Mindfulness	SAI	12.63 (4.17)	10.37 (3.34)	-2.26 (4.93)
Guidance	SMS	82.00 (12.39)	72.95 (12.37)	-9.06 (13.96)

*SMS, State Mindfulness Scale; SAI, State Anxiety Scale*.

For the participants who liked the ongoing mindfulness guidance while coloring, seventeen (out of 17) displayed an increase in state mindfulness, and sixteen (out of 17) showed a decrease in anxiety. On the other hand, the majority of participants who disliked the ongoing mindfulness guidance while coloring displayed a decrease in anxiety (14 out of 19), while state mindfulness showed to decline also (16 out of 19).

## General Discussion

Overall, results are contradicting previous findings on unguided coloring in experiment one, and introduce an advanced concept of developing mindfulness coloring books in experiment two. In experiment one, the findings suggest that there are no differences between mandala coloring and free-drawing in reducing anxiety. Furthermore, even though the majority of *mandala coloring books* and allied research have been suggested to be *mindfulness coloring books*, this first study does not support such claims. Indeed, no differences between mandala coloring and free-drawing were found in state mindfulness levels.

The differences in anxiety levels observed after the manipulation through the two conditions may be explained through several ways. First, the use of a funneled debriefing method allowed for exclusions of participants who were fully aware of the aims of the experiment, which enabled a more tightly controlled experiment. Second, the anxiety induction/manipulation that occurred in some previous coloring book investigations or the smaller sample sizes (e.g., van der Vennet and Serice, 2012; Carsley et al., 2015) may also explain the differences in findings. Third, the blindness to the experiment itself, which created an unbiased sample, which was not attracted by the popularity of coloring books, significantly improved the methodology to previous research conducted in the field. In fact, whether a self-fulfilling prophecy (or a placebo effect) is behind the use of coloring books needs further investigation, considering the subjective recruitment methods. Further research is required to draw clear conclusions, before letting coloring books come into health care settings as suggested in some academic communications (see Rigby and Taubert, 2016).

Mindfulness, has not been explored previously in association to coloring. Findings suggest that there are no differences between free-drawing and unguided coloring on state mindfulness. Findings inevitably directed the research to develop instructions on how to color mindfully, and ongoing guidance during coloring to assimilate the instructions that are usually given during mindfulness meditation as a method of increasing mindfulness through the use of coloring books.

In experiment two, teaching participants how to color mindfully, and provide continuous guidance during coloring that conforms to the instructions that are usually given during mindfulness meditation was tested as a method of increasing mindfulness through the use of coloring books, but this was not the case until liking and disliking of the guidance to color mindfully was not accounted for. Having an aversion, or, finding the practice intolerable, is indeed another judgment, that participants should have had the ability to let go off (an attitude that is expected and practiced in mindfulness meditation), which was clearly not the case in this experiment. Enabling participants to see the benefits from the first session may yet be another challenge that requires more effort and thought from mindfulness researchers. But then again, findings suggest that willingness and fondness of practices have a vast effect on outcome attainment, which brings about the question of whether we should force people into practices that are not enjoyed and pursued, whether it is mindfulness itself or mindfulness-guided coloring books. Future research should investigate naïve samples and their fondness to mindfulness practices, as the ability to adhere and upkeep those practices, and the potential of positive outcomes may be dependent upon the liking of those practices. Investigating alternative methodologies such as providing guidance prior to the activity (see e.g., Hanley et al., 2015), or utilizing longitudinal methodologies may be other ways to move this research forward. The ability of utilizing coloring as an advocate or initiation of mindfulness practices, considering the current public engagement with coloring, may be beneficial for community health.

The findings, however, may be explained through the presence of flow, and the immersion onto the task that was disrupted by the meditation teacher who was talking in the background to guide participants to color mindfully. Flow is considered a state whereby the full attentional capacity is used to the extent where there is no attention available to think about anything irrelevant (Csikszentmihalyi, 1990). The focus is only on the behavior at hand, without much awareness of the environment, surroundings, or other unrelated activities. In the present experiment, after experiencing some moments of silence, the sudden initiation of guiding/informing/advising people who are coloring may have created a disruptive and unconventional environment to immerse oneself fully. Again, future research should explore changes in flow while coloring with and without mindfulness guidance, as the benefits of unguided coloring may be more relevant to flow, rather than open awareness and mindfulness.

### Limitations

Both the use of students and the small sample size suggest that (a) caution should be used when interpreting the results, and (b) future research with a larger and more diverse sample is required. In addition, the prolonged and repetitive use of coloring books has not been investigated, and may well indicate different findings. Also, the participants consisted mostly of females. Future research should explore male participants, and the possible role of masculinity in lowering the benefits of coloring books for this part of the population; although explorations around gender during the first study did not signify any differences and did not deviate the original results reported. Another limitation that should be accounted for in future research is the use of the original state scale (see Spielberger, 1983) to enhance the reliability of the findings. Last, the random recruitment toward more Caucasian participants, and the high number of female participants in the second experiment may create a bias that needs further exploration in future research, although past explorations were primarily based on Caucasian samples and research with children revealed that males and females did not differ in anxiety reduction when coloring mandalas (Carsley et al., 2015). Another limitation that needs to be considered is the lack of a control group. The ability to separate and explain whether mindfulness-based coloring was effective because of the guidance or the coloring can only be explained by incorporating a mindfulness meditation group into a future experiment. At present, the feasibility of mindfulness coloring books entail the use of guidance on ‘how to color’ mindfully, but also require the desirability of participants to receive instructions during the coloring session.

Considering the popularity and extensive use among members of the public, it is useful to acknowledge that the use of unguided coloring books as a method of stress-reduction, mindfulness and health, and furtherance of wellbeing may depend on individual differences, and especially traits that are relevant to mindfulness, flow and rumination, which have not been explored in contemporary research. Overall, the use of coloring books may be another steadfast method of initiating and developing a mindfulness practice routine, or may become another tool to be used in contemporary secular mindfulness programs. For now, it is safe to assume that the use of guided coloring is a novel tool and research path to develop a more mindful coloring experience, and enable the augmentation of attentional training and open awareness.

## Ethics Statement

The study was approved by the Ethical Review Board of the University, and was in accordance with the ethical standards of the institutional and/or national research committee, and with the 1964 Helsinki Declaration and its later amendments. Informed written consent was obtained prior to the experiment. This study was carried out in accordance with the recommendations of the British Psychological Society. The protocol was approved by the Business, Law and Social Sciences Faculty Ethics Committee at Birmingham City University.

## Author Contributions

MM conceived the ideas, developed the interventions and discussed the proceedings and design of the research with KG. MM prepared the first draft, which was discussed and developed with KG.

## Conflict of Interest Statement

The authors declare that the research was conducted in the absence of any commercial or financial relationships that could be construed as a potential conflict of interest. The reviewer LS and handling Editor declared their shared affiliation.
